# Effect of metal/dielectric substrates on photopolymerization of BITh thin films

**DOI:** 10.1038/s41598-022-23243-4

**Published:** 2022-11-09

**Authors:** L. Hesami, C. Yang, E. Anwar, N. Noginova, M. A. Noginov

**Affiliations:** Center for Materials Research, Norfolk State University, Norfolk, VA 23504 USA

**Keywords:** Materials science, Materials for optics

## Abstract

We have studied effects of metal–dielectric substrates on photopolymerization of [2,2ʹ-Bi-1H-indene]-1,1ʹ-dione-3,3ʹ-diyl diheptanoate (BITh) monomer. We synthetized BITh and spin-coated it onto a variety of dielectric, metallic, and metal–dielectric substrates. The films were exposed to radiation of a UV–Visible Xe lamp, causing photo-polymerization of monomer molecules. The magnitude and the rate of the photo-polymerization were monitored by measuring the strength of the ~ 480 nm absorption band, which existed in the monomer but not in the polymer. Expectedly, the rate of photo-polymerization changed nearly linearly with the change of the pumping intensity. In contrast with our early study of photo-degradation of semiconducting polymer P3HT, the rate of photo-polymerization of BITh is getting modestly higher if the monomer film is deposited on top of silver separated from the monomer by a thin insulating MgF_2_ layer preventing a charge transfer. This effect is partly due to a constructive interference of the incident and reflected light waves, as well as known in the literature effects of metal/dielectric substrates on a variety of spectroscopic and energy transfer parameters. At the same time, the rate of photopolymerization is getting threefold larger if monomer is deposited on Ag film directly and charge transfer is allowed. Finally, Au substrates cause modest (~ 50%) enhancement of both monomer film absorption and the rate of photo-polymerization.

## Introduction

### Control of physical phenomena with engineered photonic environments

The research field of nanophotonics is aimed at the study of light-matter interaction at nanoscale. While control of incident light is one of the most common directions of the nanophotonics research^1–3^, the other areas include control of spontaneous^4,5^ and stimulated^6–10^ emission, Förster energy transfer^11–16^, van der Waals interactions (wetting)^17–19^, and chemical reactions^20–23^. In particular, it was shown that metallic and lamellar metal/dielectric substrates affect the rate of photodegradation and the rate of the spectral blue shift in semiconducting polymeric films P3HT (poly(3-hexylthiophene-2,5-diyl)). Importantly, it was shown that the oxidation reaction becomes accelerated if P3HT is deposited onto a metallic substrate directly and gets inhibited if the metal and the polymer are separated by a dielectric (electrically insulating) thin film of MgF_2_. While the former phenomenon was explained in terms of the charge transfer and conventional chemical catalysis, the latter effect calls for a better understanding of the underlying light-matter interaction. In this study, we ask the question whether the proximity to metallic and metal/dielectric substrates can control solid state photopolymerization, another chemical reaction of fundamental and practical importance, and compare the experimental results with the predictions of the developed theoretical model.

### Photopolymerization

The research field of photopolymerization continuously grows in both academic and industrial environments^24^. Thus, the development of photopolymerization related technologies enables new implementations in rapid prototyping, tooling, dentistry, microfluidics, biomedical devices, tissue engineering, drug delivery, etc.^25^. Commonly, photopolymerization employs monomers that can be polymerized, via radical or ionic mechanisms, in presence of photoinitistors, upon exposure to UV, visible or NIR light^24^.

C*rystalline* polymers are of importance in chemistry, physics, and materials science because they enable a wide range of advanced applications^26–28^. Fabrication of thin films of polymer crystals via spin-coating remains a challenge in polymer science^29^, as polymers tend to form amorphous phases because of entanglement of long and flexible backbones. Topochemical polymerization, a process whereby the confinement and pre-organization of the solid state forces a chemical reaction to proceed with a minimum amount of atomic and molecular movement, has provided a promising solution to the problem.

### Chemical and spectroscopic properties of BITh

**Figure 1:**
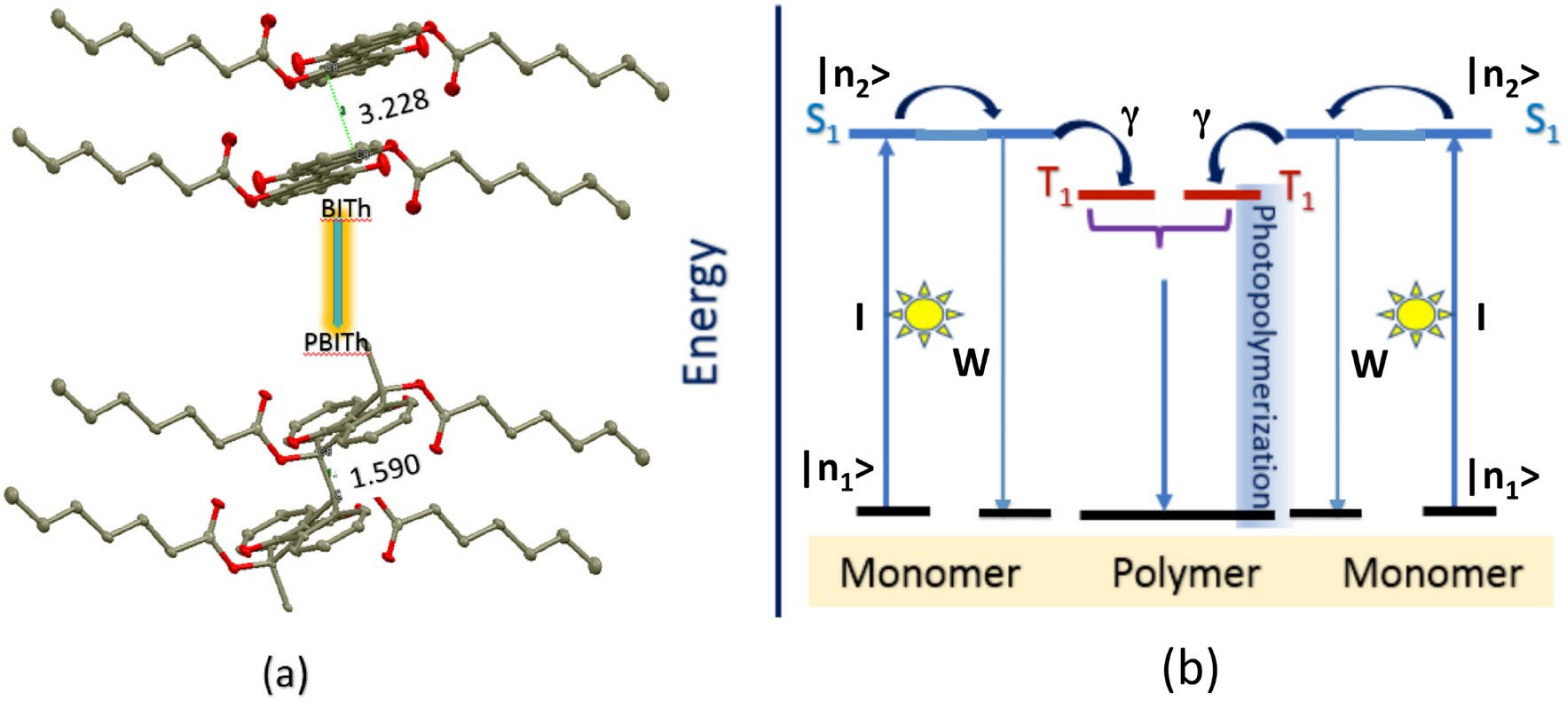
(**a**) Molecular stacking of BITh and PBITh in the crystal structure^30^. (**b**) Mechanism of photopolymerization of BITh: *S*_*1*_ singlet excited state of BITh, *T*_*1*_ triplet excited state of BITh, *I* is the pumping intensity, *W* is the rate of intracentral relaxation, and *γ* is the rate of photopolymerization, see “Modeling of photopolymerization kinetics” section^30^.

One way to study the effect of metallic and metal/dielectric substrates on photopolymerization, is to deposit a thin monomer film onto a substrate, photo-expose it causing polymerization, and monitor the reaction by measuring spectroscopic properties or optical responses, which evolve in the course of the experiment. To this end, topochemical polymerization reaction of conjugated dye molecules based on 3,3ʹ-dihydroxy-1H,1ʹH-[2,2ʹ-biindene]-1,1ʹ-dione (BIT) is an ideal system for the study^30^. [2,2ʹ-Bi-1H-indene]-1,1ʹ-dione-3,3ʹ-diyl diheptanoate (BITh) is one of thoroughly studied systems that polymerize upon UV–Visible light illumination in the solid state^26–28^. The long alkyl chains are found to play an important role in the molecular packing and, hence, topochemical reactivity (Fig. 1a).

The polymerization mechanism of BITh is believed to be similar to that reported for the diene compounds^31^. Thin film of BITh can be prepared by spin-coating from its chloroform solution. It has an absorption band ranging from ≈ 350 to ≈ 530 nm, with the maximum at ≈ 480 nm, and orange coloration. After UV or visible light illumination, the coloration and the ≈ 480 nm absorption band disappear and a colorless polymeric PBITh thin film is attained. Therefore, one can easily monitor the polymerization process of BITh using absorption and reflection spectroscopy.

The ≈480 nm spectral band is due to the π-electron delocalization (π → π*) and intramolecular donor–acceptor interactions (n → π*)^31–34^. The polymerization of BITh proceeds through a biradical and is not a simple free radical polymerization. Light absorption by a monomer leads to the singlet excited state (*S*_1_), which is a mixed state of ππ* and nπ* with intramolecular charge transfer character, thus relatively long lived. The energy of *S*_1_ can transfer from one monomer to another, or via intersystem crossing to lower triplet state (*T*_1_), Fig. 1b. Analogous to photochromic trans-syn-3,3ʹ-diaryl-2,2ʹ-biindenylidene-1,1ʹ-diones^35^, the triplet state *T*_1_ of BITh is believed to be a localized biradical that couples with lattice phonons^31–33^. This exciton-phonon coupling generates a lattice distortion, which provides a trap for the neighboring monomer molecules with *T*_1_ excited state to initiate the photoreaction (Fig. 1b). This situation would be analogous to diacetylene polymerization where the propagating species is a carbene and not a free radical^31–33,35^. Therefore, the absorption band with the maximum at ≈ 480 nm is responsible for the topochemical reactivity.

## Modeling of photopolymerization kinetics

Let us first consider a monomer film deposited on glass. Its transmission *T* is given by

1 $$T = I_{out} /I_{in} = \exp \left( { - Kl} \right) = \exp \left( { - \sigma n_{1} l} \right){, } \Rightarrow$$

2 $$K = - \ln (T)/l,$$

where *I*_*in*_ and *I*_*out*_ are incident and output light intensities, *K* = *σn*_1_ is the absorption coefficient, *σ* is the absorption cross section, *l* is the film’s thickness, and *n*_1_ is the ground state concentration of monomer molecules. Note that *T*, *l* and *K* are routinely measured experimental parameters. In this model, we neglect insignificant reflection at dielectric-dielectric and dielectric-air interfaces. We assume that the monomer has a ground state |1 > and an excited state |2 > and that the total concentration of monomer molecules *N* is equal to *N* = *n*_*1*_ + *n*_2_ ≈ *n*_1_ (*n*_2_ <  < *N*).

The rate equation for the excited state concentration *n*_*2*_ is as follows

3 $$\frac{{dn_{2} }}{dt} = \frac{{P\left[ {\text{W}} \right]}}{{S\left[ {{\text{cm}}^{2} } \right]\left( {\hbar \omega } \right)\left[ {\text{J}} \right]}}K\left[ {{\text{cm}}^{ - 1} } \right] - n_{2} W - n_{2} \gamma = I\sigma n_{1} - n_{2} W - n_{2} \gamma {,}$$

where *P* is the pumping power, *S* is the cross section area of the pumping beam, $\hbar \omega$ is the photon energy, *W* is the rate of intra-central relaxation, *γ* is the rate of photopolymerization (monomer → polymer energy transfer), and

4 $$I\left[ {{\text{s}}^{ - 1} \;{\text{cm}}^{ - 2} } \right] = \frac{{P\left[ {\text{W}} \right]}}{{S\left[ {{\text{cm}}^{2} } \right]\left( {\hbar \omega } \right)\left[ {\text{J}} \right]}},$$

is the incident pumping intensity. The system of rate equations for the concentrations *n*_1_, *n*_2_, and *N* is as follows

5 $$\frac{{dn_{2} }}{dt} = I\sigma n_{1} - n_{2} W - n_{2} \gamma ,$$

6 $$\frac{{dn_{1} }}{dt} = - I\sigma n_{1} + n_{2} W,$$

7 $$\frac{dN}{{dt}} = \frac{{dn_{1} }}{dt} + \frac{{dn_{2} }}{dt} = - n_{2} \gamma .$$

Its solution for *n*_*1*_ is given by a sum of two exponents

8 $$n_{1} = C_{1} \exp \left( { - \Lambda_{1}^{{}} t} \right) + C_{2} \exp \left( { - \Lambda_{2} t} \right),$$

where

9 $$\Lambda_{1}^{{}} \approx \left( {I\sigma + \left( {W + \gamma } \right)} \right)\quad \left[ {\text{fast decay}} \right],$$

10 $$\Lambda_{2}^{{}} \approx \frac{I\sigma \gamma }{{\left[ {I\sigma + \left( {W + \gamma } \right)} \right]}}\quad \left[ {\text{slow decay}} \right],$$

11 $$C_{1}^{{}} \approx \frac{{I\sigma + \Lambda_{2}^{{}} }}{{\Lambda_{2}^{{}} - \Lambda_{1}^{{}} }}N,\,{\text{and}}$$

12 $$C_{2} \approx \frac{{I\sigma + \Lambda_{1} }}{{\Lambda_{1} - \Lambda_{2} }}N.$$

At *W* >  > *Iσ*, *γ*,

13 $$\Lambda_{2}^{{}} \approx \frac{I\sigma \gamma }{W} = \frac{I\gamma }{W}\left( {\frac{K}{{n_{1} }}} \right) = \frac{I\gamma }{{n_{1} W}}\left( { - \ln (T)/l} \right),\; \Rightarrow$$

14 $$\frac{\gamma }{W} = \frac{{n_{1} }}{I}\frac{{\Lambda_{2}^{{}} }}{{\left( { - \ln (T)/l} \right)}} = \frac{{n_{1} }}{I} \times \left[ {Slope \, of \, \Lambda_{2}^{{}} \, vs \, \left( { - \ln (T)/l} \right)} \right],$$

where the experimental parameters *T* and *n*_1_ are evaluated before the photoexposure (*t* = 0).

We now assume that the monomer film is deposited onto a mirror-like metallic substrate, and the reflected light interferes, constructively or destructively, with the incident light. If the film thickness *l* in the reflection experiment is much smaller than the wavelength and if the film absorbs only a small fraction of the incident light (which is approximately the case of our experiment) then the sample’s reflection is given by

15 $$R = I_{out} /I_{in} = \exp \left( { - KlZ} \right) = \exp \left( { - \sigma n_{1} lZ} \right){, } \Rightarrow$$

16 $$K = \sigma n_{1} = - \ln \left( R \right)/\left( {lZ} \right),$$

where the effective absorption coefficient *K* depends on *Z*, which is the interference factor ranging between 0 (at full destructive interference) and 4 (at full constructive interference) [Z = 1 in the absence of a mirror, and Z = 2 if the wave interference is neglected].

In the photopolymerization experiment taking place on top of a highly reflective metal-based substrate, the rate equation for d*n*_2_/d*t* can be written as

17 $$\frac{{dn_{2} }}{dt} = \frac{ZP\left[ W \right]}{{S\left[ {{\text{cm}}^{2} } \right]\left( {\hbar \omega } \right)\left[ J \right]}}K\left[ {{\text{cm}}^{ - 1} } \right] - n_{2} W - n_{2} \gamma = ZI\sigma n_{1} - n_{2} W - n_{2} \gamma {.}$$

Following the derivation of Eqs. (5) to (14) and replacing *I* with *ZI*, one obtains

18 $$\Lambda_{2} = \frac{ZI\sigma \gamma }{W} = \frac{ZIK\gamma }{{Wn_{1} }} = \frac{ZI\gamma }{{Wn_{1} }}\left( {\frac{ - \ln (R)}{{lZ}}} \right) = \frac{I\gamma }{{n_{1} W}}\left( { - \ln (R)/l} \right),$$

similar to that calculated for a transparent metal-less substrate, Eq. (14). Correspondingly,

19 $$\frac{\gamma }{W} = \frac{{n_{1} }}{I}\frac{{\Lambda_{2} }}{{\left( { - \ln (R)/l_{R} } \right)}} = \frac{{n_{1} }}{I} \times \left[ {Slope \, of \, \Lambda_{2} \, vs \, \left( { - \ln (R)/l} \right)} \right].$$

Note that *K* in Eq. (18) is given by Eq. (16), and the factor *Z* cancels in the numerator and the denominator.

According to the literature, the values *K*^36^, *σ*^37^, *γ*^14^ and *W*^38^ on top of metal can be different from those on top of glass or MgF_2_. This is the subject of the study discussed below.

## Synthesis, fabrication, and spectroscopic studies

### Synthesis of [2,2ʹ-Bi-1H-indene]-1,1ʹ-dione-3,3ʹ-diyl diheptanoate (BITh)

**Figure 2:**
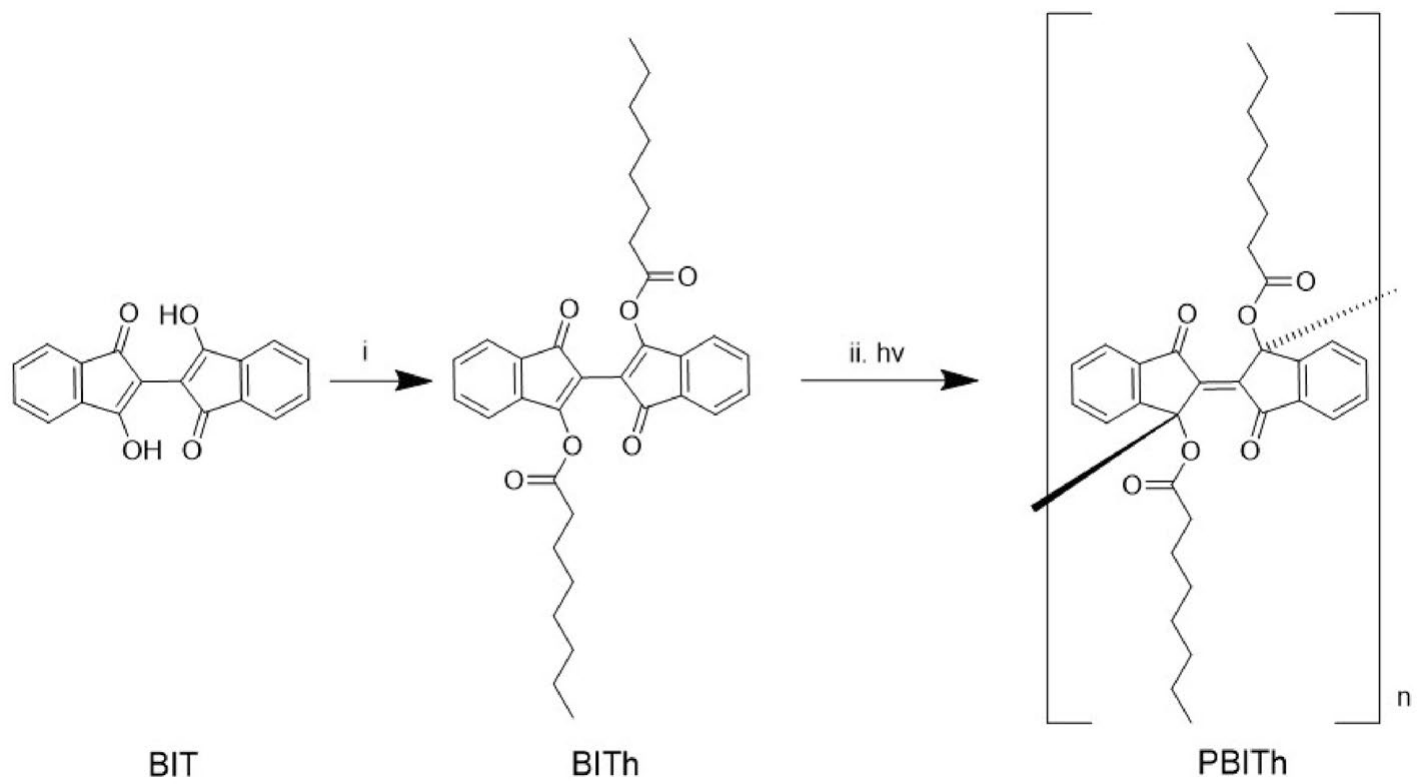
Synthesis of monomer and polymer. Reaction conditions: (i) heptanoyl chloride, triethylamine, dichloromethane, 0 °C to room temperature, 5 h; (ii) ħν: where ħ is Planck’s constant, and ν is the frequency of light^30^.

The monomer BITh was synthesized, with modification, according to the procedure described by Dou et al.^30^ and Gabriel^39^, by functionalizing BIT with heptanoate on the hydroxyl groups in the 3 and 3ʹ positions (Fig. 2). In a 100 mL two-neck round-bottom flask, 3,3ʹ-dihydroxy-1H,1ʹH-[2,2ʹ-biindene]-1,1ʹ-dione (BIT, 150 mg) was dissolved in 20 mL dry dichloromethane (freshly distilled over sodium) under argon protection. The mixture was cooled in an ice bath to 0 °C, and 0.42 mL triethylamine (redistilled over sodium) was added. To the resulting dark red solution, heptanoyl chloride (0.33 mL) was added dropwise at 0 °C. After addition, the solution was stirred for 30 min at 0 °C. The resulting orange-red solution was stirred at room temperature overnight. 20 mL of water was added to quench the reaction. The organic part was separated, washed with water, dried over MgSO_4_ and then passed through a short silica gel with CH_2_Cl_2_ as eluent. The solution was concentrated to ~ 5 mL using rotary evaporator, filtered, and 20 mL ethanol was added to it. The resulting mixture was standing overnight and the red precipitate was filtered and washed with ethanol (3 × 10 mL: until ethanol washings colorless) and dried in an oven at ~ 125 °C for 10 min to obtain red crystalline solid. Recrystallization of EtOH-CH_2_Cl_2_ resulted in fiber-like crystals (60 mg, yield 25%). BITh (10 mg) was then dissolved in 1.0 mL freshly distilled CH_2_Cl_2_ or CHCl_3_ to give an orange red solution, which was spin-coated onto the glass substrates. Knowing the molar weight (*M* = 542.7 g/mol)^30^ and the density (*ρ* = 1.294 g/cm^3^)^30^ of BITh, the molecular concentration of solid BITh was evaluated to be equal to *N* = 1.44 × 10^21^/cm^3^.

### Experimental samples and setups

Experimentally, we have fabricated and studied BITh monomer thin films (23–90 nm) deposited on (1) glass, (2) MgF_2_ on top of glass, (3) Ag on top of glass, (4) MgF_2_ on top of Ag deposited on glass, (5) lamellar Ag/MgF_2_ structure deposited on glass (with MgF_2_ as the top layer), and (6) Au on top of glass. Silver, gold and MgF_2_ were deposited using the thermal vapor deposition technique (Nano 36 apparatus from Kurt J Lesker) and BITh monomer was spin coated (using the Spin Coater from Specialty Coating System) onto the substrates listed above. The thickness of the fabricated organic and inorganic films was measured using the stylus DekTak XT profilometer from Bruker.

The samples were illuminated with the Xe lamp, model OPS-A150 from Oriel. The power P_Σ_, irradiated in the whole spectrum (ranging from UV to mid-infrared), was measured with the powermeter from Scientech (model 67005, the sensor area 7.1 cm^2^). The power radiated by the Χe lamp into the λ ~ 480 nm absorption band of the monomer was calculated as

20 $$P_{\Delta \lambda } = P_{\Sigma } \frac{{\int {\Omega \left( \lambda \right)K\left( \lambda \right)} d\lambda }}{{K_{\max } \int {\Omega \left( \lambda \right)} d\lambda }},$$

where *Ω(λ)* was the emission spectrum of the lamp^40^, *K(λ)* was the monomer’s absorption spectrum, and *K*_*max*_ was the maximal monomer’s absorption coefficient.

**Figure 3:**
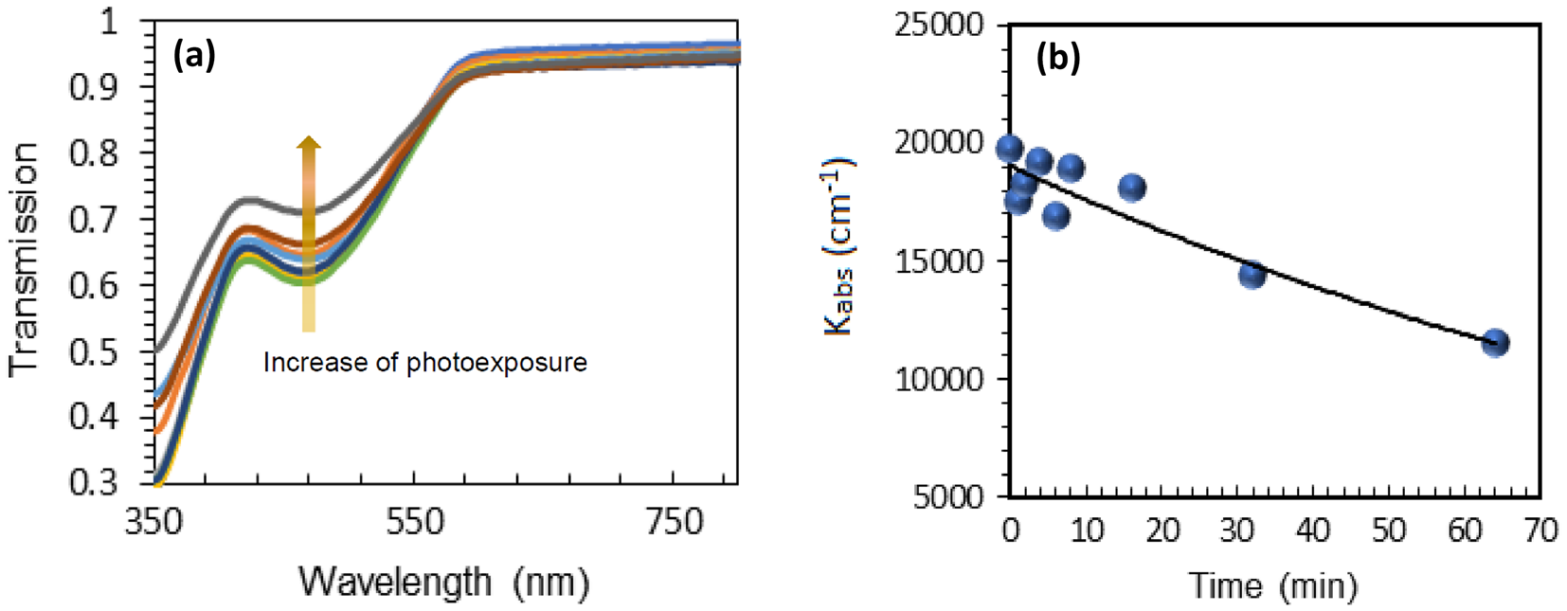
(**a**) Transmission spectra of the BITh film after photoexposures ranging from 0 to 64 min. (**b**) Characters: Kinetics of the maximal absorption coefficients in the photoexposed BITh film. Solid line: fit with the exponential function.

The samples were photoexposed over one-to-thirty min, and the integral photoexposure time ranged between 1 and 70 min. After each photoexposure, the sample’s transmission spectra (on top of transparent substrates) or reflection spectra (on top of metal-based substrates) were taken using Lambda 900 Spectrophotometer from PerkinElmer. The spectra were further normalized by the transmission or reflection of the substrates. After the absorption backgrounds were properly subtracted, the transmission and reflection spectra showed the dips centered at λ ~ 480 nm, which were due to the monomer’s absorption. With increase of the photoexposure, the dips became more shallow, manifesting photopolymerization of the monomer, Fig. 3a. When the strengths of the corresponding absorption bands were plotted against the photoexposure time, the resultant polymerization kinetics could, in the first approximation, be described with the exponential functions, Fig. 3b.

The strengths of the monomer’s absorption bands and the rates of their photodegradation, measured on top of different substrates, are the major experimental results of this study as discussed below. Most of the monomer solutions and thin film samples were synthesized, fabricated and experimentally studied two or three times. In those cases, the average values of the measured parameters were calculated and used in the data analysis.

## Experimental results and discussion

Monomer BITh molecules have absorption band at ~ 480 nm^30^, while polymerized BITh molecules do not. Therefore, photopolymerization is accompanied by photobleaching and change in transmission and reflection spectra with increase of the photoexposure time and the radiation fluence, Fig. 3a. The dependence of the ≈ 480 nm experimental absorption coefficient (determined as $K^{\exp } = - \ln \left( T \right)/l$ in the transmission experiment and $K^{{{\text{exp}}}} = - \ln \left( R \right)/l$ in the reflection experiment) on the exposure time resulted in the absorption kinetics depicted in Fig. 3b.

When BITh molecules were spin coated onto MgF_2_ films deposited on top of glass substrates, the maximal absorption coefficient before the photoexposure (determined as $K^{\exp } = - \ln \left( T \right)/l$) was equal to 2.03 × 10^4^/cm. Given the concentration of BIT molecules to be equal to *N* = 1.44 × 10^21^/cm^3^, the maximal absorption cross section is equal to *σ* = 1.41 × 10^–17^ cm^2^. The nearly similar result was obtained when the BITh monomer was spin coated on a glass slide directly.

In most of photoexposure experiments, the power radiated by the Xe lamp within the *λ* ~ 480 nm absorption band of the monomer was equal to *P*Δ*λ* = 0.10 W (Eq. (20)). The corresponding intensity was equal to *I* = 3.4 × 10^16^/cm^2^/s, and the pumping rate was equal to *Iσ* = 0.49/s.

As Λ_2_, *K*^*exp*^ = − ln(*T*)/*l*, *I*, and *n*_1_≈*N* are known, one can evaluate the ratio *γ*/*W* to be 2.8 × 10^–4^. Assuming that *W* ~ 10^9^/s (typical rate of S_1_ → S_0_ transition in organic dye molecules^41^), *γ* ≈ 10^5^/s (This is in agreement with “Modeling of photopolymerization kinetics” section, where we assumed that both *Iσ* and *γ* are much smaller than *W*).

**Figure 4:**
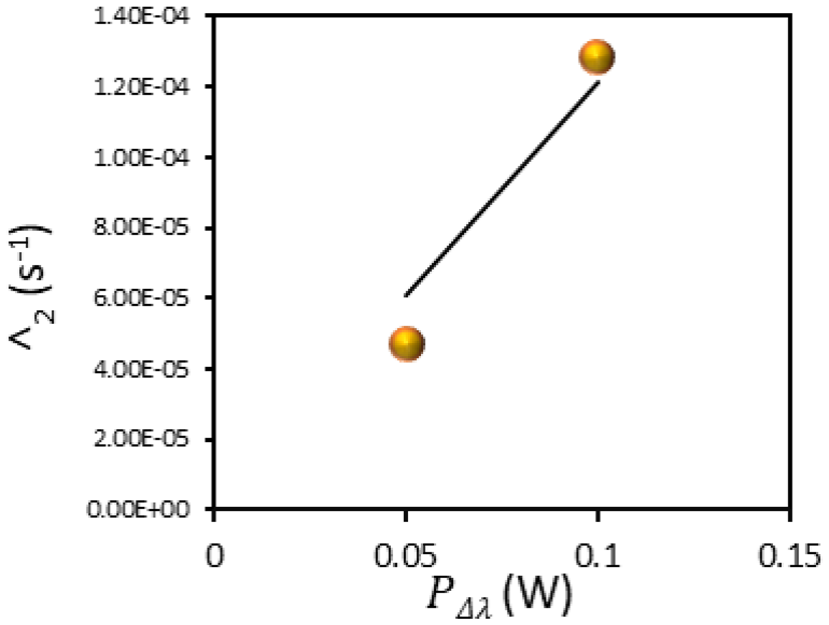
Nearly linear dependence of the decay rate Λ_2_ on the excitation power PΔλ.

In the next particular experiment, *P*Δ*λ* was reduced twofold (by increasing the distance between the lamp and the glass/MgF_2_/BITh sample) and the measured value Λ2 decreased almost two times (Fig. 4), in agreement with the theoretical prediction (Eq. (13)).

**Figure 5:**
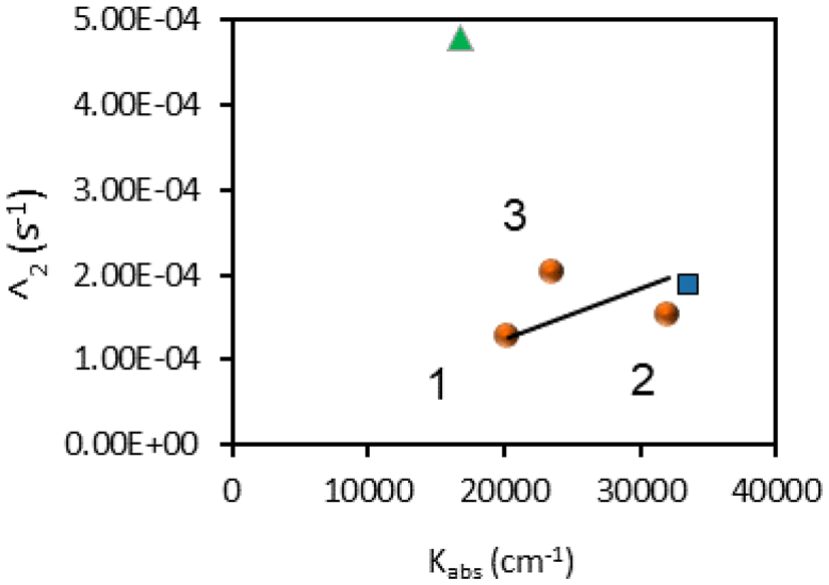
The decay rate *Λ*_2_ plotted against the absorption coefficient $K^{\exp } = - \ln \left( R \right)/l$ (or $K^{\exp } = - \ln \left( T \right)/l$). Circle 1: glass/MgF_2_/BITh; circle 2: glass/Ag/MgF_2_/BITh; and circle 3: glass/Ag–MgF_2_ lamellar structure/BITh. Triangle—BITh deposited on Ag. Square—BITh deposited on Au.

In the two experimental samples discussed next, (i) glass/Ag/MgF_2_/BITh and (ii) glass/Ag–MgF_2_ lamellar structure/BITh, the monomer was separated from Ag by a thin insulating MgF_2_ layer. In these samples (circles 2 and 3 in Fig. 5), the data points (*Λ*_2_, − ln(*R*)/*l*) were reasonably close to the corresponding data point in the glass/MgF_2_/BITh metal-free sample, circle 1 in Fig. 5. Furthermore, the three circle character data points (two in the samples with metal and one in the sample without metal) formed a straight line, although with a notable data scatter, Fig. 5. This suggests that the ratio *γ*/*W* (determined by the slope *Λ*_2_ *vs* − ln(*R*)/*l*) was practically not affected by Ag separated from BITh molecules with a thin insulating MgF_2_ film. At the same time, the values *Λ*_2_ and − ln(*R*)/*l* in the Ag based samples (circles 2 and 3) were marginally larger than the corresponding point, *Λ*_2_ and (− ln(*T*)/*l*), in the metal-free MgF_2_ sample, circle 1 in Fig. 5. This effect can be explained by a partial constructive interference of incident and reflected light waves in Ag-based samples, increasing both the effective absorption $K^{\exp } = - \ln \left( R \right)/l$ (or $K^{\exp } = - \ln \left( T \right)/l$) and the decay rate *Λ*_2_.

At the same time, in the glass/Ag/BITh samples, in which monomer molecules were not separated from Ag by an insulating layer, the decay rate *Λ*_2_ was nearly threefold higher than that in the samples with MgF_2_ layer. A similar effect, observed in Peters et al.^20^ photodegradation of the semiconducting polymer P3HT, was tentatively explained by a metal-polymer charge transfer (chemical catalysis).

Lastly, note that the Au substrate caused modest (~ 50%) enhancement of both monomer film absorption (− ln(*R*)/*l*) and the decay rate *Λ*_2_, in comparison to BITh on top of MgF_2_ without metal, Fig. 5. The detailed study of the effect of Au-based substrates on photopolymerization of BIT is the subject of a separate study to be published elsewhere.

## Summary

We have studied effects of metal-dielectric substrates on photo-polymerization of [2,2ʹ-Bi-1H-indene]-1,1ʹ-dione-3,3ʹ-diyl diheptanoate (BITh) monomer. Experimentally, we synthetized BITh and spin-coated it onto a variety of dielectric, metallic, and metal-dielectric substrates. The films were exposed to radiation of a UV–Visible Xe lamp, causing photo-polymerization of monomer molecules. The magnitude and the rate of the photo-polymerization were monitored by measuring the strength of the ≈ 480 nm spectral band, which existed in the monomer but not in the polymer. Before photo-exposure, the absorption coefficient of BITh deposited onto MgF_2_ film was equal to 20,300/cm. Irradiation of the monomer with the light intensity 0.014 W/cm^2^ (in the spectral band of the BITh absorption) caused its polymerization (reduction of the ≈ 480 nm absorption band) occurring at the rate of 1.28 × 10^–4^/s. Expectedly, the rate of photo-polymerization changed nearly linearly with the change of the pumping intensity. In contrast with Peters et al.^20^, the rate of photo-polymerization is getting modestly higher if the monomer film is deposited on top of silver separated from monomer by a thin insulating MgF_2_ layer preventing charge transfer. This effect is partly due to constructive interference of the incident and reflected light waves. However, the latter interference is not the only decisive factor determined the rate of photo-polymerization, as W^38^, σ^37^, and γ^14^ are known to be affected by the vicinity of metal/dielectric interfaces. This is the subject of the further studies. At the same time, in agreement with Peters et al.^20^, the rate of photopolymerization is getting threefold larger if monomer is deposited on Ag film directly and charge transfer is allowed. Finally, Au substrates cause modest (~ 50%) enhancement of both monomer film absorption and rate of photo-polymerization, the phenomenon to be studied and published elsewhere.

## Data Availability

The datasets used and/or analysed during the current study available from the corresponding author on reasonable request.
